# Altered Mental Status in a 16-Year-Old Adolescent Male

**DOI:** 10.7759/cureus.11664

**Published:** 2020-11-23

**Authors:** Dahima Cintron, Jean C Rodríguez-Agramonte, Leslie A Soto-Velez

**Affiliations:** 1 Internal Medicine - Pediatrics, University of Puerto Rico, Medical Sciences, San Juan, PRI; 2 Pediatric Hematology - Oncology, University of Puerto Rico, Medical Sciences, San Juan, PRI

**Keywords:** hemaphagocytosis, macrophage activation, emergency, rheumatology, hematology

## Abstract

We report a case of a 16-year-old Hispanic male, without history of systemic illness, who presented with altered mental status and fevers since two weeks prior to evaluation. Further history revealed one-month complaints of headaches, nocturnal fevers, right knee and elbow pain, fatigue, loss of appetite, transient finger discoloration, and a nine-pound weight loss. Physical exam was remarkable for a thin male with pale mucosa, petechia on palate and distal extremities, malar rash that included nasal bridge and cervical and posterior lymphadenopathy. Laboratory work-up showed pancytopenia, with elevated ferritin value of 11,320 ng/mL. The patient was diagnosed with juvenile-onset systemic lupus erythematosus (JSLE) with macrophage activation syndrome (MAS) and suspected antiphospholipid syndrome (APS). Our patient’s predominant presentation were neurological symptoms. These can be seen in up to one-third of patients with MAS. They can range from headache, seizures, altered mental status, irritability, and lethargy. Other symptoms are fevers, lymphadenopathy, and hepatosplenomegaly. Ferritin values above 10,000 are highly specific and sensitive for MAS. Albeit a more common presentation in juvenile idiopathic arthritis, MAS can also present across other auto-immune diseases.

## Introduction

Macrophage activation syndrome (MAS) is a rheumatological emergency within hemophagocytic lymphohistiocytosis (HLH), that is termed MAS when it occurs secondary to autoimmune or infectious diseases. The clinical manifestations can vary and involve multiple systems, including the central nervous system. This report documents the case of an adolescent boy with neurological symptoms as main manifestations of MAS secondary to juvenile onset systemic lupus erythematosus (JSLE). A literature search was conducted to explain the patient's clinical presentation and understand the current limitations in diagnosis of MAS among pediatric patients and the current management recommendations.

## Case presentation

A healthy 16-year-old Hispanic male, a resident of Texas, presented to the Emergency Department with one day of altered mental status. In the last month, he complained of headaches after a soccer head collision. The headaches were intermittent, throbbing, lasted less than five minutes, and were associated with nausea and dizziness. Albeit visits to a concussion clinic and oral anti-inflammatory drugs, he persisted with headaches and developed nocturnal fevers (37.8 C), right knee and elbow pain, fatigue, loss of appetite, transient finger discoloration and a nine-pound weight loss in the last 30 days. Head CT and right-hand doppler performed by concussion clinic where found with no abnormalities. He arrived at the Caribbean two weeks ago, where fevers became refractory to antipyretics. The rest of the review of systems was noncontributory, including recent exposure to risk factors such as camping trips or wild animal contact.

At initial presentation, he was febrile (38.6 C) with pulse of 72 beats per minute, respiratory rate of 20 breaths/min, blood pressure of 113/75 mmHg and adequate saturation at room air. He appeared thin and frail with pale mucosa, petechia on palate and distal extremities, and malar rash that includes nasal bridge. Cervical and posterior lymphadenopathy was present with unremarkable cardiac and lung exam. No oral/nasal ulcers or swollen joints were found. He had no motor or sensory deficits but was disoriented and somnolent, not answering or following commands. He did not have nuchal rigidity, and cranial nerves and deep tendon reflexes were intact.

A complete blood cell count showed a white blood cell count of 2.52 uL, with bandemia of 18%, hemoglobin of 9 g/dL, hematocrit value of 26.1%, mean corpuscular volume of 83.7, and platelet concentration of 63 uL. Anemia work-up showed hemolysis with a positive Coombs test with low reticulocyte count of 0.42%, elevated lactate dehydrogenase of 982 U/L and total bilirubin normal at 0.42 mg/dL. Creatinine level was 1.51 mg/dL and blood urea nitrogen (BUN) of 39 mg/dL suggesting an acute kidney injury. Labs were also remarkable for coagulation tests showing normal international normalized ratio (INR) at 1.03 and prothrombin time (PT) at 11.7 seconds, but prolonged partial thromboplastin time (PTT) at 68.9 seconds. Ferritin level was elevated at 11,329 ng/mL. No hematuria or proteinuria was found on urine analysis.

Our patient was admitted for close neuro-checks and further work-up with broad differential extending from infectious vs malignancy vs autoimmune. Head CT was negative for cerebrovascular accidents. Given the patient's endemic state is Texas, Lyme disease and ehrlichiosis were considered as well as other arbovirus, fungal and bacterial etiologies. The first lumbar puncture was a dry tap and a second attempt was not performed due to high risk of bleeding. He was started on intravenous broad-spectrum antibiotics and hydration. Overnight neuro-checks showed improvement in mental status, with patient answering questions and following simple commands. A brain MRI found acute/subacute ischemic infarcts and areas of narrowing of the bilateral M3 and M4 branches. Hematology service was consulted due to pancytopenia who orders inflammatory markers shown in Table 1 that confirmed a rheumatological emergency.

**Table 1:** Serological results meeting diagnostic criteria met for macrophage activation syndrome based on the 2016 Ravelli classification criteria [1].

Ferritin	11,329 ng/mL
Triglycerides	125 mg/dL
Aspartate Aminotransferase	114 u/L
Fibrinogen	190 mg/dL
Platelet count	63 x10^9 ^/liter

**Figure 1:**
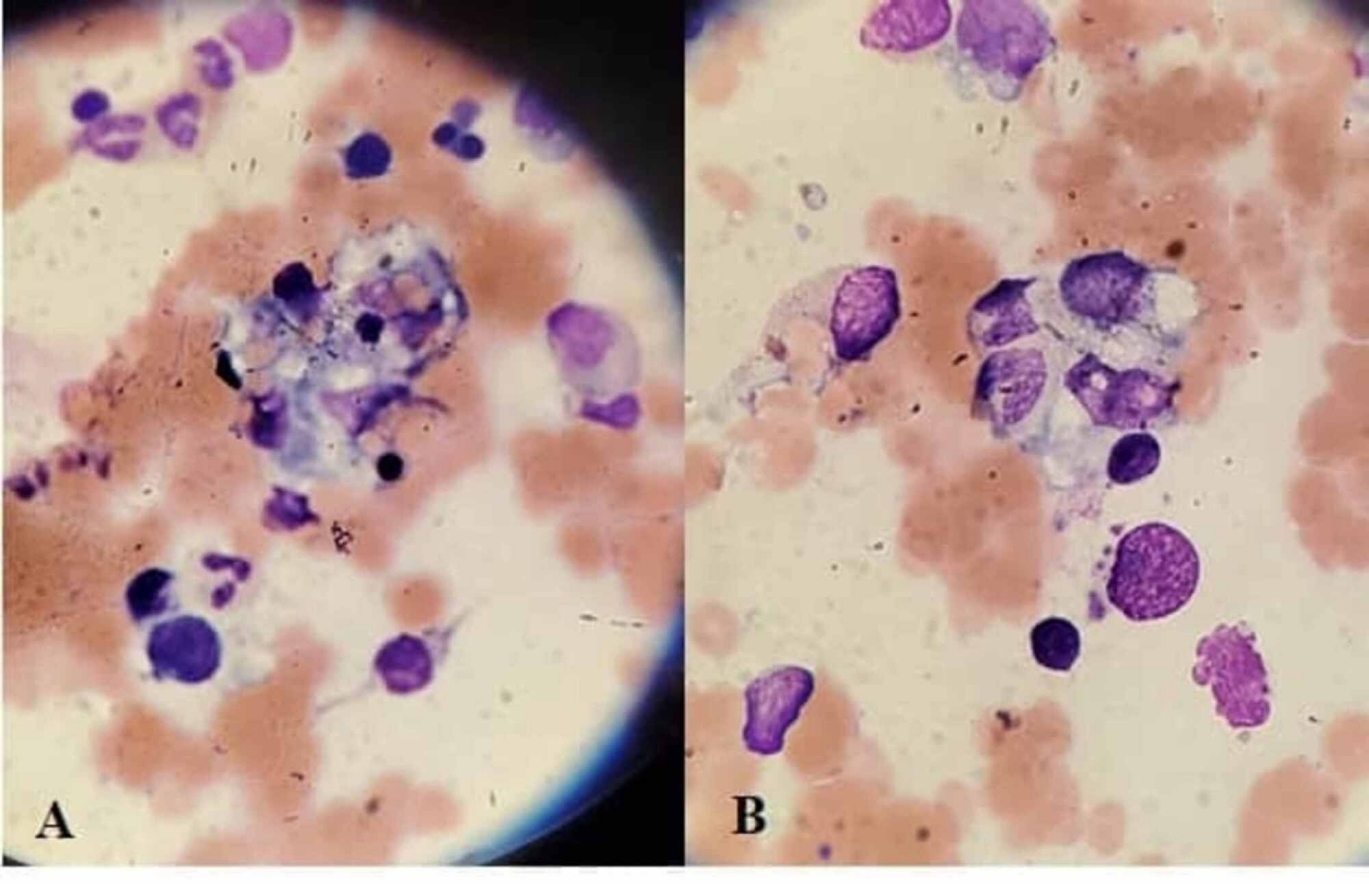
Microscopic examination of the bone marrow shows numerous well-differentiated macrophages actively phagocytosing hematopoietic cells.

Further questioning highlighted a strong maternal family history of autoimmune disease. He has a maternal aunt with Hashimoto's thyroiditis and ongoing work-up for systemic lupus erythematosus, while his maternal grandmother had rheumatoid arthritis. Given the high clinical suspicion patient was given intravenous methylprednisolone pulses for three days, followed by a daily dose (3mg/kg/d) and hydroxychloroquine 300mg daily. A bone marrow biopsy was performed, as shown in Figure 1, which together with a ferritin level of 11,239 and further serological tests shown in Table 2, confirmed the diagnosis. Remaining infectious and rheumatological serological work-up, including flow cytometry tests, returned negative. Soluble interleukin-2 receptor (IL-2R) or natural killer cell function assays were not obtained. 

**Table 2:** Serological results meeting immunological criteria for Juvenile onset systemic lupus erythematosus with suspected concomitant anti-phospholipid syndrome according to 2019 European League Against Rheumatism (EULAR)/American College of Rheumatology (ACR) criteria [2] and 2006 Sydney Criteria [3]. IgG: immunoglobulin G, DNA: deoxyribonucleic acid, CRP: C-reactive protein, ANA: antinuclear antibody

ANA	Positive (1: 640, homogeneous)
Anti-double stranded DNA	Positive (27)
C3	Low (27)
C4	Low (3)
Anti-Cardiolipin IgG	Positive (15)
Lupus Anticoagulant	Positive (Detected)
D- Dimer	5.84
Erythrocyte sedimentation rate	107
CRP	23
Lactate Dehydrogenase	928

The patient was diagnosed with juvenile onset systemic lupus erythematosus (JSLE) with macrophage activation syndrome (MAS) and suspected antiphospholipid syndrome (APS). One week later the cutaneous and hematological abnormalities persisted, and IV cyclophosphamide was started. Cylclophosphamide regime consisted of a dose 500mg/m2 every four weeks for a six-month period. He was evaluated by ophthalmology service for transient vision loss history, with findings of right eye peripheral vascular occlusion. Given suspected APS with a thrombo-embolic event, he was started on low molecular weight heparin for a minimum of three months. 

## Discussion

MAS can present across all age ranges, with estimates of 10% complicating juvenile idiopathic arthritis (JIA) and lower across other immune conditions such as SLE ranging from 0.9 to 4.6% [4]. Although the pathogenesis remains unclear, and genetic predisposition may play a role, it is postulated that a massive systemic inflammation, marked by prolonged cytotoxic T cells and macrophage activation, results in elevated cytokine levels ("cytokine storm") causing multi-system organ failure and death. In addition, there is macrophage activation resulting in hemophagocytosis, or engulfment, of red blood cells, white blood cells, and platelets [5]. At the moment it remains unclear if this engulfment initiates or responds to the elevated cytokines.

The clinical manifestations can vary and involve multiple systems. Our patient’s predominant presentation were neurological symptoms. These can be seen in up to one-third of patients with MAS [6]. They can range from headache, seizures, altered mental status, irritability, and lethargy. Other symptoms are fevers, lymphadenopathy, and hepatosplenomegaly [4]. Regarding laboratory results, the changes are non-specific ranging from pancytopenia, coagulopathy, and hypofibrinogenemia. An important finding is elevated ferritin levels, which are speculated to be a cytoprotective response to reduce apoptosis causes by increased oxidative stress [5]. Ferritin levels at 2,000 are concerning for HLH/MAS, while values above 10,000 are highly suggestive of the disease [7].

The diagnosis of MAS is challenging and requires high index of suspicion. First, as a secondary presentation, MAS can have different phenotypes based on the triggering factor [8]. Second, the criteria used to diagnose HLH is used interchangeably for MAS [8]. The HLH criteria are based on the primary autosomal recessive form, initially established in 2004, and later revised and edited in 2009 by Filipovich [9] (Table 3). This criterion may have some overlap with MAS, a secondary presentation, but the testing is often extensive, not specific to the patient’s acute presentation, and unpractical in many centers, prolonging diagnosis, and early treatment of MAS. 

**Table 3:** The 2009 Modified Histiocyte Society diagnostic criteria for hemophagocytic lymphohistiocytosis (HLH) [9]. ANC: absolute neutrophil count, NK: natural killer, Hgb: hemoglobin, Plt: platelet

At least 3:			
Fever	> 38.5 for more than 7 days		
Splenomegaly	3cm below costal margin		
Cytopenia involving 2 lines	Hgb < 9 g/dL	ANC < 100/ mcL	Plt < 100,00
Hepatitis	No defined threshold		
At least 1:			
Ferritin	> 500 ug/L		
CD25	> 2,400 U/ml		
Low Absent NK cell activity	Local lab reference		
Hemophagocytosis	Observed on bone marrow biopsy		
Other supportive features (not required):			
Hypertriglyceridemia	> 265 mg/dL		
Hypofibrinogenemia	< 1.5 g/L		
Hyponatremia	No defined threshold		

In view of these limitations, and the common presentation of MAS secondary to JIA the European League Against Rheumatism (EULAR)/American College of Rheumatology (ACR) developed MAS criteria for patients with JIA [1] (Table 4). At the moment, they remain the closest accepted criteria to be applied in pediatric patients with an autoimmune disorder. Our patient’s autoimmune diagnosis was SLE, and it is unclear how these criteria apply to other autoimmune presenting patients.

**Table 4:** The 2016 Ravelli Classification Criteria for diagnosis of macrophage activation syndrome in patients with juvenile idiopathic arthritis [1].

Ferritin	> 684 ng/mL
And any 2 of the following:	
Platelet count	<181 x10^9^/liter
Aspartate Aminotransferase	>48 units/liter
Triglycerides	>156 mg/dL
Fibrinogen	<360 mg/dL

Bone marrow biopsy could be a helpful diagnostic criterion, showing increased hemophagocytic activity in the bone marrow. Yet hemophagocytosis may not be present in initial stages and is neither sensitive nor specific for MAS [6].

Due to high mortality from MAS, treatment should be started as soon as there is clinical suspicion. First-line treatment is intravenous glucocorticoids, usually methylpredinosole at 30 mg/kg/dose for one to three days. If adequate response, patients are transitioned to 2-3 mg/kg/day dosing and later to oral prednisolone. For non-responders, such as our patient, cyclophosphamide can be used, at 500-750 mg/m2 intravenous monthly doses for three to six months. If refractory to previous options, other options directed for HLH can be tried. These are usually more targeted therapies such as etoposide, anti-thymocyte globulins, Interleukin 1 inhibitor, or even intravenous immunoglobulins [4].

## Conclusions

MAS is a rheumatological emergency that requires prompt medical management due to high morbidity/mortality. Currently, diagnostic criteria for MAS is unclear, available guidelines in pediatric populations are limited to the 2016 Ravelli classification created for patients with JIA, which have yet to prove diagnostic in other autoimmune diseases. Ferritin levels above 10,000 are highly suggestive of MAS. Bone marrow aspiration is part of confirmatory testing but should not delay the start of therapy once suspicion arises. Initial treatment consists of intravenous glucocorticoids. If refractory, targeted therapies used in HLH may be considered.
